# Legal

**Published:** 1881-05

**Authors:** 


### LEGAL.

Below is given the text of a law, passed February 5th, 1881, to regulate the practice of dentistry in the State of Alabama. This is one of the most rigid laws, as yet passed, bearing on this subject, and in the main its features are very good. If it is faithfully executed, quackery can neither obtain nor maintain a foothold in that State. Would that every State in the Union had such protection.

*An Act to Regulate the Practice of Dentistry in the State of Alabama.*

Section 1. Be it enacted by the General Assembly of Alabama, That from and after the passage of this act, it shall be unlawful for any person to engage in the practice of dentistry in the State of Alabama, unless said person has obtained license from a Board of Dental Examiners, duly authorized and appointed by this Act to issue such license.

Sec. 2. Be it further enacted, That the Board of Dental Examiners shall consist of five dental graduates, or practitioners of dentistry, who have obtained a license to practice dentistry from a Medical Board in this State, or from a Dental Board organized under this act, and who are members in good standing of the Alabama Dental Association; *Provided*, That said graduates or practitioners have been practicing dentistry in the State of Alabama, for a period not less than three years; And *provided further*, That the first Board of Examiners under this Act, shall consist of the present executive committee of the Alabama Dental Association, who shall hold office until the next annual meeting of the said Association, and until their successors are elected and qualified as hereinafter provided.

SEC. 3. Be it further enacted, That it shall be the duty of the said Alabama Dental Association, at its annual meeting, next after the passage of this Act, and every two years thereafter to elect said Board of Examiners, who shall hold office for the term of two years, and until their successors are elected and qualified. The President of said Association shall have power to fill all vacancies in said Board for unexpired terms.

SEC. 4. Be it further enacted, That it shall be the duty of said Board of Examiners: 1st, to meet annually at the time and place of meeting of the Alabama Dental Association, or oftener at the call of any three of the members of said Board. Thirty day's notice must be given of the time and place of meeting of said Board, said notice to be mailed to all practicing dentists in the State. 2nd, to prescribe a course of reading for those who study dentistry under private instruction. 3rd, to grant a license to any applicant who shall furnish satisfactory evidence of having graduated and received a diploma from any incorporated Dental College, or who has heretofore received a license from a Medical Board in this State without examination or fee. 4th, to grant a license to all other applicants, who undergo a satisfactory examination, who shall pay to the said Board a fee of five dollars for said license. 5th, to keep a book in which shall be registered the names of all persons licensed to practice dentistry in this State.

SEC. 5. Be it further enacted, That the book so kept, shall be a book of record, and a transcript from it certified to by the officer who has it in keeping, with the common seal of said Board, shall be evidence in any court in this State.

SEC. 6. Be it further enacted, That three members of said Board shall constitute a quorum for the transaction of business, and should a quorum not be present on the day appointed for its meeting, those present may adjourn from day to day, until a quorum is present.

SEC. 7. Be it further enacted, That one member of said Board may grant a license to an applicant to practice until the next regular meeting of the Board, when he shall report the fact, at which time the temporary license shall expire, but such temporary license shall not be granted by a member of the Board after the Board has rejected the applicant.

SEC. 8. Be it further enacted, That any person who shall, in violation of this Act, practice dentistry in this State, for a fee, or reward, shall be liable to indictment, and on conviction shall be fined not less than fifty nor more than three hundred dollars; *Provided*, That nothing in this Act shall be construed to prevent any person from extracting teeth.

SEC. 9. Be it further enacted, That on the trial of such indictment, it shall be incumbent upon the defendant to exempt him from the penalties of this Act, to show that he has authority under the law to practice dentistry in this State.

SEC. 10. Be it further enacted, That every person to whom a license is issued by said Board of Examiners, shall, within thirty days from the date thereof, present the same to the Judge of the Probate Court of the county in which he resides, who shall officially endorse said license, and seal it with the seal of the court, and who shall record said license in a proper book in his office, and who shall be entitled to receive a fee of one dollar for his services, but a temporary license issued under Section 7 of this Act, need not be sealed or recorded.

SEC. 11. Be it further enacted, That it shall be the duty of the Solicitors of this State to prosecute all persons violating all or any portion of this Act.

SEC. 12. Be it further enacted, That all laws or parts of laws in conflict with this Act be, and the same are hereby repealed.

